# Supplementary material for: The interactome of intact mitochondria by cross-linking mass spectrometry provides evidence for coexisting respiratory supercomplexes
Source: Mol Cell Proteomics. 2017 Dec 8;17(2):216–32. doi: 10.1074/mcp.RA117.000470 (PMC5795388; doi:10.1074/mcp.RA117.000470)
Supplement: Supplemental Data [file supp_RA117.000470_133922_0_supp_23978_fzffwf.zip › spectra_annotation/mito_DR_spectra_annotation/460-1-12-1-15-1.pdf]

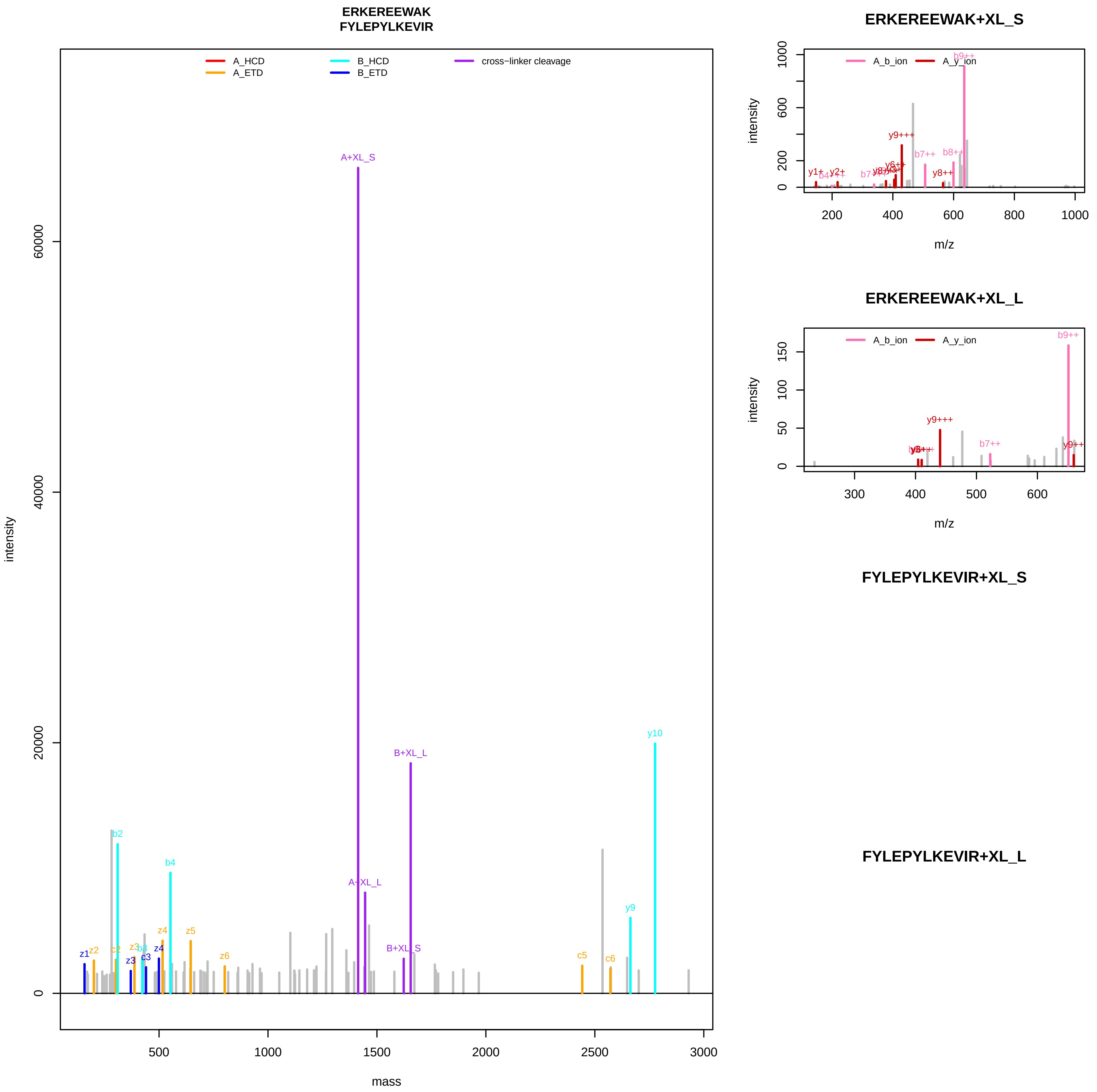

ERKEREEWAK
FYLEPYLKEVIR
A_HCD
A_ETD
B_HCD
B_ETD
cross−linker cleavage
A+XL_S
B+XL_L
A+XL_L
B+XL_S
y10
y9
b2
b4
b3
z1
z2
c2
z3
c3
z4
z5
z6
c5
c6
intensity
mass
0
20000
40000
60000
500
1000
1500
2000
2500
3000
ERKEREEWAK+XL_S
A_b_ion
A_y_ion
b9++
y9+++
b7++
b8++
y8++
y6++
y1+
y2+
intensity
m/z
0
200
600
1000
200
400
600
800
1000
ERKEREEWAK+XL_L
A_b_ion
A_y_ion
b9++
y9+++
b7++
y9++
intensity
m/z
0
50
100
150
300
400
500
600
FYLEPYLKEVIR+XL_S
FYLEPYLKEVIR+XL_L
